# Comprehensive analysis of m^6^A regulators characterized by the immune microenvironment in Duchenne muscular dystrophy

**DOI:** 10.1186/s12967-023-04301-5

**Published:** 2023-07-11

**Authors:** Xu Han, Guang Ji, Ning Wang, Le Yi, Yafei Mao, Jinliang Deng, Hongran Wu, Shaojuan Ma, Jingzhe Han, Yi Bu, Pingping Fang, Juyi Liu, Fanzhe Sun, Xueqin Song

**Affiliations:** 1 Department of Neurology, The Second Hospital of Hebei Medical University, Shijiazhuang, 050000 Hebei People’s Republic of China; 2 The Key Laboratory of Neurology (Hebei Medical University), Ministry of Education, Shijiazhuang, 050000 Hebei People’s Republic of China; 3 Neurological Laboratory of Hebei Province, Shijiazhuang, 050000 Hebei People’s Republic of China; 4 Department of Laboratory Medicine, The First Hospital of Hebei Medical University, Shijiazhuang, 050000 Hebei China; 5 Department of Neurology, Handan Central Hospital, Handan, 050000 Hebei People’s Republic of China

**Keywords:** Duchenne muscular dystrophy, Immune microenvironment, m^6^A regulators, Diagnostic signature

## Abstract

**Background:**

Duchenne muscular dystrophy (DMD) is an X-linked, incurable, degenerative neuromuscular disease that is exacerbated by secondary inflammation. N^6^-methyladenosine (m^6^A), the most common base modification of RNA, has pleiotropic immunomodulatory effects in many diseases. However, the role of m^6^A modification in the immune microenvironment of DMD remains elusive.

**Methods:**

Our study retrospectively analyzed the expression data of 56 muscle tissues from DMD patients and 26 from non-muscular dystrophy individuals. Based on single sample gene set enrichment analysis, immune cells infiltration was identified and the result was validated by flow cytometry analysis and immunohistochemical staining. Then, we described the features of genetic variation in 26 m^6^A regulators and explored their relationship with the immune mircoenvironment of DMD patients through a series of bioinformatical analysis. At last, we determined subtypes of DMD patients by unsupervised clustering analysis and characterized the molecular and immune characteristics in different subgroups.

**Results:**

DMD patients have a sophisticated immune microenvironment that is significantly different from non-DMD controls. Numerous m^6^A regulators were aberrantly expressed in the muscle tissues of DMD and inversely related to most muscle-infiltrating immune cell types and immune response-related signaling pathways. A diagnostic model involving seven m^6^A regulators was established using LASSO. Furthermore, we determined three m^6^A modification patterns (cluster A/B/C) with distinct immune microenvironmental characteristics.

**Conclusion:**

In summary, our study demonstrated that m^6^A regulators are intimately linked to the immune microenvironment of muscle tissues in DMD. These findings may facilitate a better understanding of the immunomodulatory mechanisms in DMD and provide novel strategies for the treatment.

**Supplementary Information:**

The online version contains supplementary material available at 10.1186/s12967-023-04301-5.

## Introduction

Duchenne muscular dystrophy (DMD) is an X-linked, incurable, degenerative neuromuscular disease caused by mutations in the *DMD* gene coding for dystrophin protein. The absence of dystrophin compromises the integrity of the sarcolemma and leads to uncontrolled inflammation, which is followed by extensive degeneration of the muscle fibers [1]. Currently, there is no cure for DMD, although numerous therapeutic strategies have been developed to improve survival. Glucocorticoids remain the standard of therapy, but their use is limited by the occurrence of side effects such as Cushing’s syndrome. Several promising therapeutic strategies aimed at the restoration of dystrophin production, including gene therapy and stem cell therapy, have been hampered by the few benefited population and the hosts' immune response [2–5]. Therapies designed to ameliorate inflammation in the muscle microenvironment represent a feasible therapeutic avenue to both prevent muscle deterioration and enhance the tolerability of emerging approaches [6]. Therefore, further characterization of the muscle microenvironment and extensive exploration of the immunomodulatory mechanisms is indispensable to develop effective therapies.

Compelling evidence suggests the crosstalk between the immune system and DMD [7, 8]. A previous study has identified that aberrant signaling pathways regulate immune processes leading to the degenerative process of DMD [9]. Enhanced expression of inflammatory genes and increased infiltration of activated immune cells are evident early in the progress of DMD [10, 11]. Since many unknown factors could influence the immune status, the regulatory mechanisms responsible for immunity are not fully elucidated. N^6^-methyladenosine (m^6^A) modification is the most prevalent internal transcript modification of RNA in eukaryotes, which is dynamically mediated by specific m^6^A regulatory enzymes, including “methyltransferases” (mainly METTL3 and METTL14), “reading proteins” and “demethylases” (ALKBH5 and FTO) [12]. m^6^A modification is widely involved in various physiological and pathological processes [13, 14]. Emerging evidence indicates that aberrant expression and mutation in the m^6^A regulators were related to abnormal processes, including metabolism abnormality, dysregulated cell cycle and proliferation, etc. [15, 16]. Recently, several studies have demonstrated that m^6^A regulators have a close relationship with immunological regulation [17]. For instance, the deletion of m^6^A reader YTHDF2 enhances the activation of NF-κB and MAPK signaling pathways to upregulate the expression of osteoclast-associated gene and immunity processes [18]. Besides, the m^6^A writer METTL3 facilitates M1 macrophage to M2 macrophage polarization by STAT1 methylation [19]. To our knowledge, however, few studies have explored the relationship between m^6^A modification and the immune microenvironment in DMD until now.

In this work, we studied the characteristics of the immune environment in the muscle tissues of DMD based on the next-generation sequencing data. Flow cytometry (FCM) of the muscle tissues in mdx mice (a mouse model of DMD) and immunohistochemistry (IHC) in DMD patients were introduced to validate the infiltration of dominant immune cells. Then, we performed a systematic assessment of the DMD m^6^A modification pattern and revealed the close relationship between m^6^A regulators and the immune microenvironment in the muscle tissues of DMD. In summary, our findings uncovered the potential role of m^6^A modification in the immune microenvironment of DMD and may provide new potential therapeutic avenues for this disease.

## Materials and methods

### Animals

Mdx and C57BL/6 mice were purchased from the Nanjing Biomedical Research Institute of Nanjing University (Nanjing, China). All experiments were conducted based on protocols and approved by the Second Hospital of Hebei Medical University Animal Care and Use Committee (approval number: 2022-AE283). The sample sizes of mice in the experiment were established according to previous experience and the analyses were terminated when the differences between each group were considered statistically significant [20].

### Patient samples

Human tissues were collected from patients with suspected muscle disease admitted to the Second Hospital of Hebei Medical University. All of the patients signed written informed consents to allow the collection of muscle samples and agreed to use these samples/cells for research purpose. The diagnosis of DMD was confirmed by genetics. Negative muscle samples included patients referred for muscle discomfort who had normal histology, histo-enzymology, and immunohistochemistry at the time of muscle biopsy assessment (Additional file 7: Table S1). The analyses were stopped after analyzing 4 patients’ biopsies because a clear statistical difference between DMD patients and non-DMD controls (n = 4) was observed.

### Microarray datasets collection and data process

Microarray datasets were retrieved from the Gene Expression Omnibus (GEO) database, including GSE109178, GSE6011, GSE38417, and GSE1004 [21–23]. For GSE109178 and GSE38417, the probe IDs were annotated using the platform GPL570, while GSE6011 was on the platform of GPL96. The expression dataset profile of GSE1004 was based on the GPL91 and GPL8300 platform. R package “limma” was used for background adjustment and quantile normalization. If a gene symbol corresponds to multiple probes, the average level of the expression value will be determined. Due to the small sample size could affect the power of statistical analysis and lead to inaccurate results, three datasets (GSE109178, GSE6011, and GSE38417) were integrated as a training set to expand the sample size. We selected an independent dataset GSE1004 to externally validate the gene expressions of key regulators. The Combat function of the “sav” R package was used to correct batch effects and principal component analysis (PCA) was introduced to evaluate the performance (Additional file 1: Figure S1).

### Immune characteristics analysis for the microarrays datasets

Gene set enrichment analysis (GSEA) was used to explore the potential immunological pathways by GSEA software (version 4.1.0). Single-sample gene set enrichment analysis (ssGSEA) was conducted to assess the immunocyte fractions in DMD patients. The list of genes involved in gene-sets of infiltrating immune cells was obtained from the prior study [24]. To identify the variation of biological processes between DMD and normal tissues, R package “GSVA” was introduced to run Gene set variation analysis (GSVA) enrichment analysis, and the latest version of immune response gene-sets was acquired from the platform MSigDB (http://software.broadinstitute/org/gsea/msigdb/). The Wilcox test was introduced to analyze the enrichment scores of immune response activity and immune cell abundance between muscle biopsy specimens from patients with DMD and non-DMD controls.

### Isolation of muscle leukocytes and flow cytometry analysis

Four-week-old male C57BL/6 and mdx mice were euthanized via cervical dislocation, and the muscles from mouse limbs were harvested and rinsed in cold saline. Muscle tissues were then prepared for single-cell suspension by mesh rubbing method. Briefly, muscles were placed on a 150-mesh sieve, washed with saline three times, and a 25 mL small beaker was placed under the sieve. Then, the tissues were cut into pieces, rinsed with saline and collected in the beaker. The mixture was then filtered and a 300-mesh nylon sieve was used to remove cell debris followed with centrifuged at 157*g* for 5 min. We collected and re-suspended the pellet, layered it on an equal volume of Lymphocyte separation media (MultiSciences, Hangzhou, China), and centrifuged at 400*g* for 20 min. The interface of cells was collected, re-suspended in 4 ml of saline, and centrifuged at 157*g* for 5 min. The supernatants were discarded and the rest were re-suspended with saline. The following antibodies used for staining were purchased from MultiSciences (Hangzhou, China): APC-Cy7-anti-CD3, APC-anti-F4/80, FITC-anti-CD4, PE–anti-CD45, PE-Cy7-anti-CD11b, and PerCP-Cy5.5–anti-CD8. Optimal working dilutions were determined according to the relevant protocol. All antibodies were incubated for 30 min at 4 ℃. All flows were done using FACS ARIA II (BD Biosciences) and the data were analyzed using FlowJo 8.2.6 (Tree Star, Ashland, OR).

### Histopathological and immunohistochemical (IHC) assay

The muscle biopsy specimens from DMD patients and non-DMD controls were freshly frozen in liquid nitrogen–cooled isopentane. The frozen muscle Sects. (8 μm) were stained with HE and pathological changes were observed under a light microscope. IHC assay was according to the previous manufacturer's suggestion [25]. Briefly, the dry slides were preblocked in PBS containing 10% normal goat serum and incubated overnight with the primary antibodies for macrophages (rat monoclonal anti-mouse F4/80 antibody, Abcam, Cambridge, UK), CD4 positive T cells (mouse monoclonal antibody against CD4, Maxim, Fuzhou, China) or CD8 positive T cells (mouse monoclonal antibody against CD8, Maxim, Fuzhou, China), respectively. Then, the cells were rinsed and incubated with the appropriate secondary antibody (Proteintech, Wuhan, China) at 20 ℃ for 20 min. 3,3-diaminobenzedine tetrahydrocloride (Solarbio, Beijing, China) was used as chromogenic substrate. Lastly, the cells were counterstained with haematoxylin and mounted. The Ab binding was observed under a microscope.

### Identification of m^6^A regulators

27 widely recognized m^6^A RNA methylation regulators were collected from published literatures. These regulators including 9 writers (CBLL1,METTL3, METTL5, PCIF1, RBM15, RBM15B, WTAP, ZC3H13, and ZCCHC4), 2 erasers (ALKBH5 and FTO), and 16 readers (ELAVL1, EIF3A, FMR1, G3BP1, G3BP2, HNRNPA2B1, HNRNPC, IGF2BP2, IGF2BP3, LRPPRC, PRRC2A, YTHDC1, YTHDC2, YTHDF1, YTHDF2, and YTHDF3.). The online platform of String (cBioportal) (http://www.string-db.org/) and Cytoscape were utilized to evaluate protein–protein interaction (PPI). The correlation between m^6^A RNA methylation regulators was performed by R package “corrplot” (*P* < 0.05 as cut-off criteria).

### Differential analysis of m^6^A regulators

Differentially expressed m^6^A regulators were performed by R package “limma”. Univariate logistic regression was introduced to determine m^6^A regulators in DMD patients (*P* < 0.05 as cut-off criteria). Least absolute shrinkage and selection operator (LASSO) regression was applied for minimizing the overfitting. Then the refined regulators were used to establish a predicting model. According to the coefficients obtained from the LASSO, the risk score equals the sum of coefficients and m^6^A regulator expression values. Receiver operating characteristic (ROC) curve analysis and the area under the ROC curve (AUC value) were finally used to evaluate the distinguishing performance.

### Correlation analysis between m^6^A regulators and immune characteristics

Spearman correlation analyses were conducted to evaluate the relevance between m^6^A regulators and infiltrating immunocytes populations, immune response activity, and HLA gene expression. Heatmap was used for visualizing the results.

### Unsupervised clustering for m^6^A regulators

By unsupervised clustering analysis, diverse m^6^A modification patterns were identified according to the expression profiles of m^6^A regulators. The cluster numbers and robustness were assessed by consensus clustering. We ran 1000 iterations of the above steps to guarantee the classification robustness with the R package “ConsensuClusterPlus”. The m^6^A modification patterns were further validated through PCA.

### Biological pathway analysis

Kyoto Encyclopedia of Genes and Genomes (KEGG) pathway enrichment and HALLMARKS pathway were introduced to identify relevant enriched biological pathways in distinct m^6^A modification patterns. The expression matrix was transformed into the pathway activation score matrix through GSVA. Raw P values were corrected for multiple testing using the false discovery rate (FDR) and the thresholds were set at FDR < 0.05. Gene Ontology (GO) enrichment analysis was applied to access the major biological functions of m^6^A phenotype-associated genes by the R package “clusterProfiler” and adjusted *P* < 0.01 was considered as the cut-off criterion.

### Identification of m^6^A regulator-mediated genes

To determine m^6^A regulator-mediated genes, R package “limma” was performed to identify differential expression genes (DEGs) between distinct m^6^A modification patterns. We overlapped the DEGs to determine the m^6^A phenotype-associated genes and visualized the result with Venn plot.

### Statistical analyses

R (version 3.6.1) and SPSS (version 25.0) were introduced to perform data analysis and statistics. Student's t-test or Mann–Whitney U-test was carried out to compare differences between two independent groups. One-way ANOVA or Kruskal–Wallis tests were performed for the comparisons among three or more groups. Spearman correlation analysis was used to identify the relevance of gene expression. |R|> 0.25 and *P* < 0.05 were considered relevant and identified as statistically significant unless otherwise mentioned.

## Result

### Characteristics analysis of immune microenvironment in DMD

**Fig. 1:**
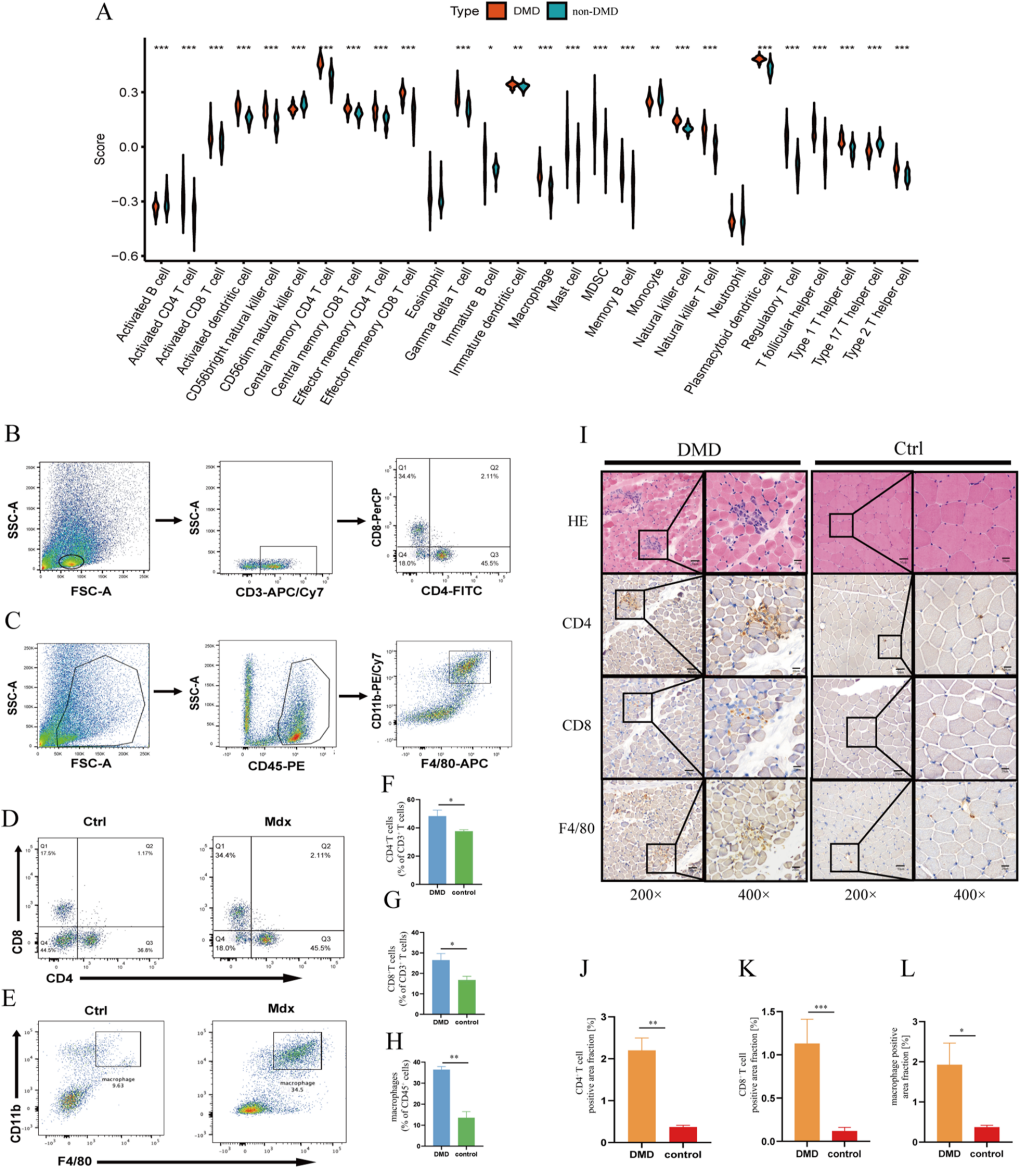
Characteristics analysis of the immune microenvironment in DMD. **A** Violin plots show the distributions and levels of immune cell infiltration (DMD: non-DMD = 56:26). **B** and **C** Schematic of gating strategy of flow cytometry analysis. **D** and **E** Representative flow cytometry profiles shows CD4^+^, CD8^+^, and CD11b^+^F4/80^Hi^ macrophages in the skeletal muscle of mdx and C57 mice. **F**, **G**, and **H** Cell populations are quantified as percentage of the total number of immune cell population; (F and G) distribution of CD4^+^ and CD8^+^ T cells (%CD3^+^) (n = 4); (H) distribution of CD11b^+^F4/80^Hi^ macrophages (%CD45^+^) (n = 3). **I** Cross sections of skeleton muscles were hematoxylin and eosin stained or immunohistochemically stained either with a mouse monoclonal antibody against CD4 or CD8 for T cells or a rat monoclonal antibody F4/80 antibody to identify macrophages. Dark-brown colored cells represent 3, 3-diaminobenzidine tetrahydrochloride positive macrophages or T cells. **J**, **K** and **L** Infiltration of CD4^+^ T cells, CD8^+^ T cells or macrophages (n = 4) was quantified in three random microscopic fields using a 20 × objective. Scale bar, 50 μm. *P < 0.05; **P < 0.01; ***P < 0.001

The main immune-related biological processes and molecular functions associated with the pathogenesis of DMD were investigated by GSEA. The results suggested that many significant immune response-associated processes might be involved in the pathology of DMD, such as antigen progression and presentation, complement and coagulation cascades, and leukocyte transendothelial migration (Additional file 2: Figure S2A–F). Furthermore, we found 20/23 immune reaction related pathways significantly upregulated in DMD compared with non-DMD samples, indicating enhanced immune responses of muscle tissues in DMD (Additional file 3: Figure S3A and S3B, Tables S2). By utilizing ssGSEA method, we explored infiltrating immune cells difference between DMD and non-DMD groups and found that the extent of immune infiltration was significantly higher in DMD group (P < 0.05, Fig. 1A, Tables S3). Furthermore, we conducted FCM of the skeleton muscle in mdx and C57BL/6 mice to preliminarily validate the immune-cell infiltration status in DMD (Fig. 1B, C). As shown in Fig. 1D–H, the proportions of CD4^+^, CD8^+^ T cells and macrophages are significantly increased in mdx compared with the control group. Similarly, IHC staining for CD4^+^ T cell (CD4), CD8^+^ T cell (CD8) and macrophage (F4/80) using muscle samples of DMD patients and non-DMD controls validated the results (Fig. 1I). The staining signals for T cells and macrophages in DMD groups were notably higher than non-DMD control (Fig. 1J–L). In addition, we explored the HLA gene expression status and found that most of them were altered in DMD compared with non-DMD controls (Additional file 4: Figure S4, Tables S4).

### The landscape of m^6^A regulators in DMD

**Fig. 2:**
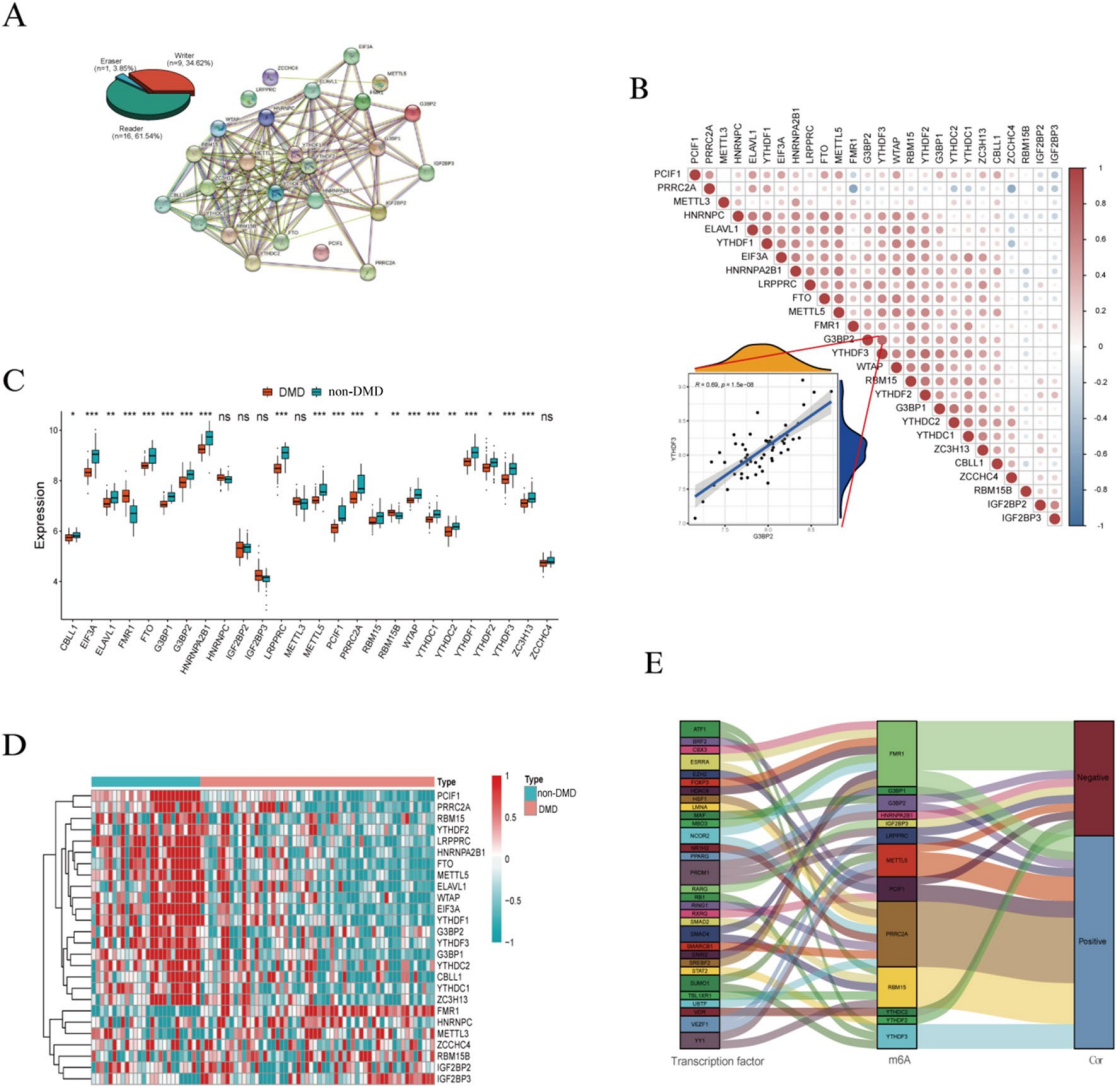
Expression level of m^6^A regulators in DMD. **A** (left panel) The top pie chart represents the proportion of writers, readers and erasers. (right panel) A PPI network showing the interaction between m^6^A regulators. **B** The correlation matrix reflects the correlations among m^6^A regulators. **C** and **D** The box-plot and heatmap indicate the expression values of m^6^A regulators between DMD and non-DMD samples. **E** Alluvial diagram showing the relevance between transcription factors and m^6^A regulators. *P < 0.05; **P < 0.01; ***P < 0.001; ns, no significance

To explore the potential role of m^6^A regulators in the immune environment of DMD, we evaluated the expression pattern of m^6^A regulators. 26 m^6^A regulators were selected for analysis in our work, including 9 writers, 16 readers, and 1 eraser (Additional file 7: Table S5). The results of PPI network indicated that m^6^A regulators had a tight association and functioned as a complex (Fig. 2A). Meanwhile, the correlation among 26 m^6^A regulators at transcription levels was analyzed and the results indicated a strong relationship exists between m^6^A regulators. Among them, G3BP2-YTHDF3 showed the most significant positive correlations with *R* = 0.69 (Fig. 2B). We further explored differential expression levels of 26 m^6^A regulators between DMD and non-DMD controls and 21 regulators were found to be significantly altered in the muscle tissues of DMD patients, including 19 down-regulated genes and 2 up-regulated genes (Fig. 2C and D). The expression values of 6 writers (CBLL1, ZC3H13, METTL5, RBM15, WTAP, and PCIF1), 12 readers (ELAVL1, HNRNPA2B1, LRPPRC, YTHDC1, YTHDC2, YTHDF1, YTHDF2, YTHDF3, PRRC2A, G3BP1, EIF3A, and G3BP2), and 1 eraser (FTO) were reduced, whereas the expression values of reader FMR1 and writer RBM15B were significantly increased (*P* < 0.05). Among these differentially expressed regulators, PCIF1 showed the most statistically significant alteration and FMR1 showed the maximum fold-change in DMD (Additional file 7: Table S6). To elucidate the possible regulatory mechanism of m^6^A methylation modification, we analyzed the relationship between transcription factors and m^6^A regulators in DMD. Based on correlation coefficients greater than 0.6, 31 transcription factors associated with the m^6^A regulators were identified (Additional file 7: Table S7). As shown in Fig. 2E, there is a complex relation between m^6^A regulators and transcription factors. Among them, PRRC2A (reader), RBM15 (writer), and FMR1 (reader) were associated with diverse transcriptional factors; PRRC2A and RBM15 showed a significant positive correlation with most of factors, while FMR1 showed a negative correlation. In summary, our data identified aberrant expression levels of m^6^A methylation regulators and showed the complexity of gene regulation through m^6^A modification mechanisms in DMD patients.

### Identification of key m^6^A regulators in DMD

**Fig. 3:**
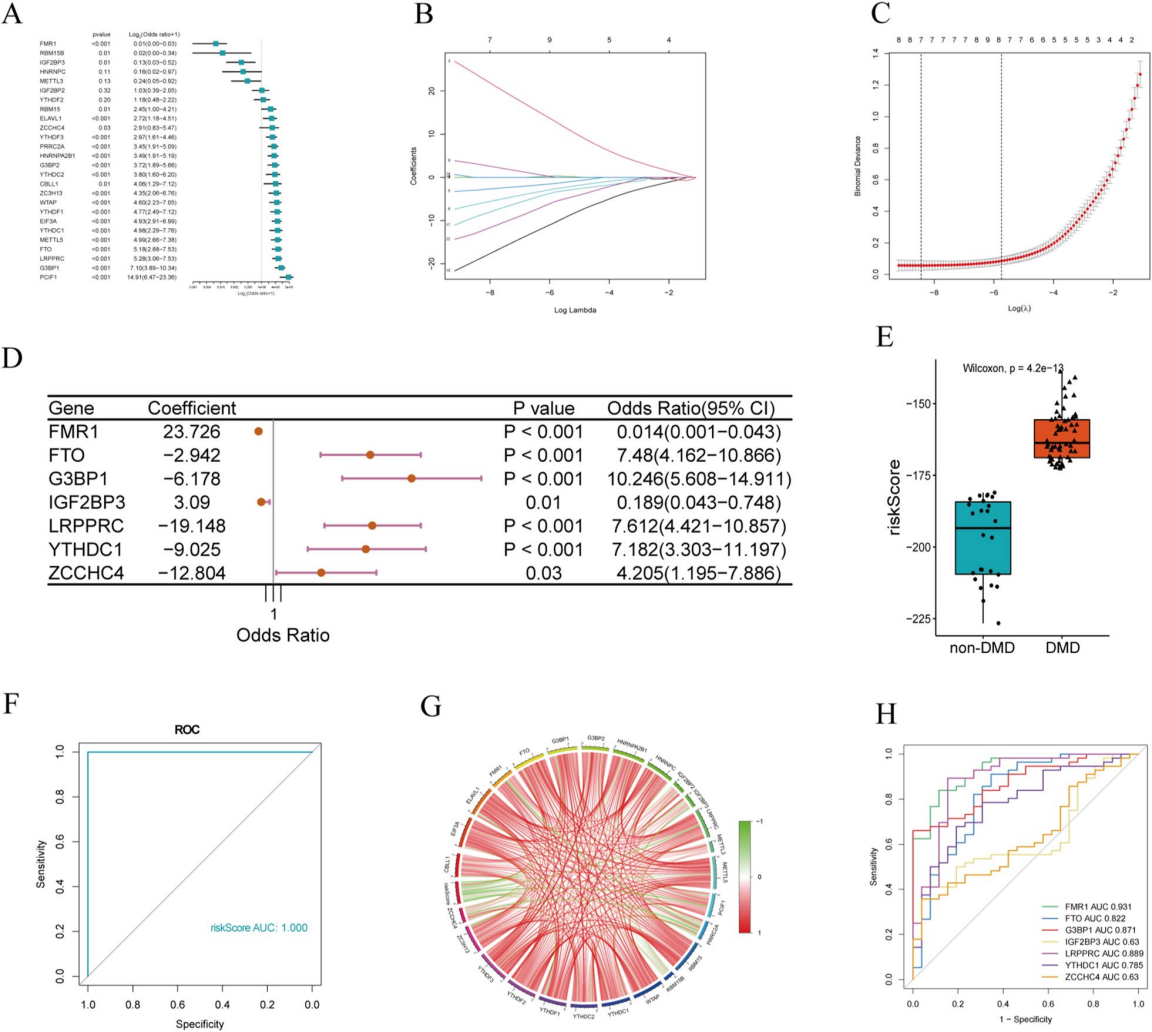
LASSO logistic regressions of the m^6^A-related signature. **A** Univariate logistic regression analysis of DMD for 26 m^6^A related regulators, and 22 genes with *P* < 0.05. **B** LASSO coefficient profiles of a model featuring the selected seven genes. **C** LASSO analysis with minimal lambda value. **D** Multivariate logistic regression analysis reveals the distinguishing signature with seven m^6^A regulators. **E** The risk distribution between non-DMD and DMD. **F** ROC curves for the 7 m^6^A regulators diagnostic model. **G** A map exhibits the relevance between risk score and m^6^A regulators. **H** ROC curves for the seven m^6^A genes. AUC, the area under the ROC curve. *P < 0.05; **P < 0.01; ***P < 0.001

The univariate logistic regression analysis was performed to determine critical m^6^A factors in DMD. Our result revealed that 22 m^6^A regulators were related to the development of DMD (Fig. 3A, Additional file 7: Table S8). Then, LASSO regression was introduced to avoid overfitting in the subsequent model construction (Fig. 3B, C). According to the optimum λ value, 7 genes (FMR1, FTO, G3BP1, IGF2BP3, LRPPRC, YTHDC1, and ZCCHC4) were selected as hub m^6^A regulators for DMD, which were then applied to construct a gene signature (Additional file 7: Table S9). Then we performed a logistic multifactor regression analysis (Fig. 3D) and calculated the diagnostic risk score of the gene signature to reveal its ability in distinguishing between normal and DMD samples. As is shown in Fig. 3E, the DMD group experienced a higher m^6^A risk score than the control group. The ROC curve analysis also suggested that the gene signature has a good performance in classifying the two groups (AUC = 1, Fig. 3F). Furthermore, the relationship between risk score and 26 m^6^A regulators in DMD samples was investigated. The risk score was negatively associated with most regulators, whereas positively linked to FRM1 and RBM15B (Fig. 3G). Moreover, a ROC curve of 7 m^6^A regulators was performed to estimate the accuracy of the candidate genes and the AUCs for these regulators ranged from 0.63 to 0.931 (Fig. 3H), indicating our results’ high accuracy. We selected an independent dataset GMS1004 from the GEO database to externally validate the gene expressions of key regulators. The result shows a similar tendency to the training set, which can prove the reliability of our analysis (Additional file 5: Figure S5).

### The relevance between m^6^A regulators and immune characteristics in DMD

**Fig. 4:**
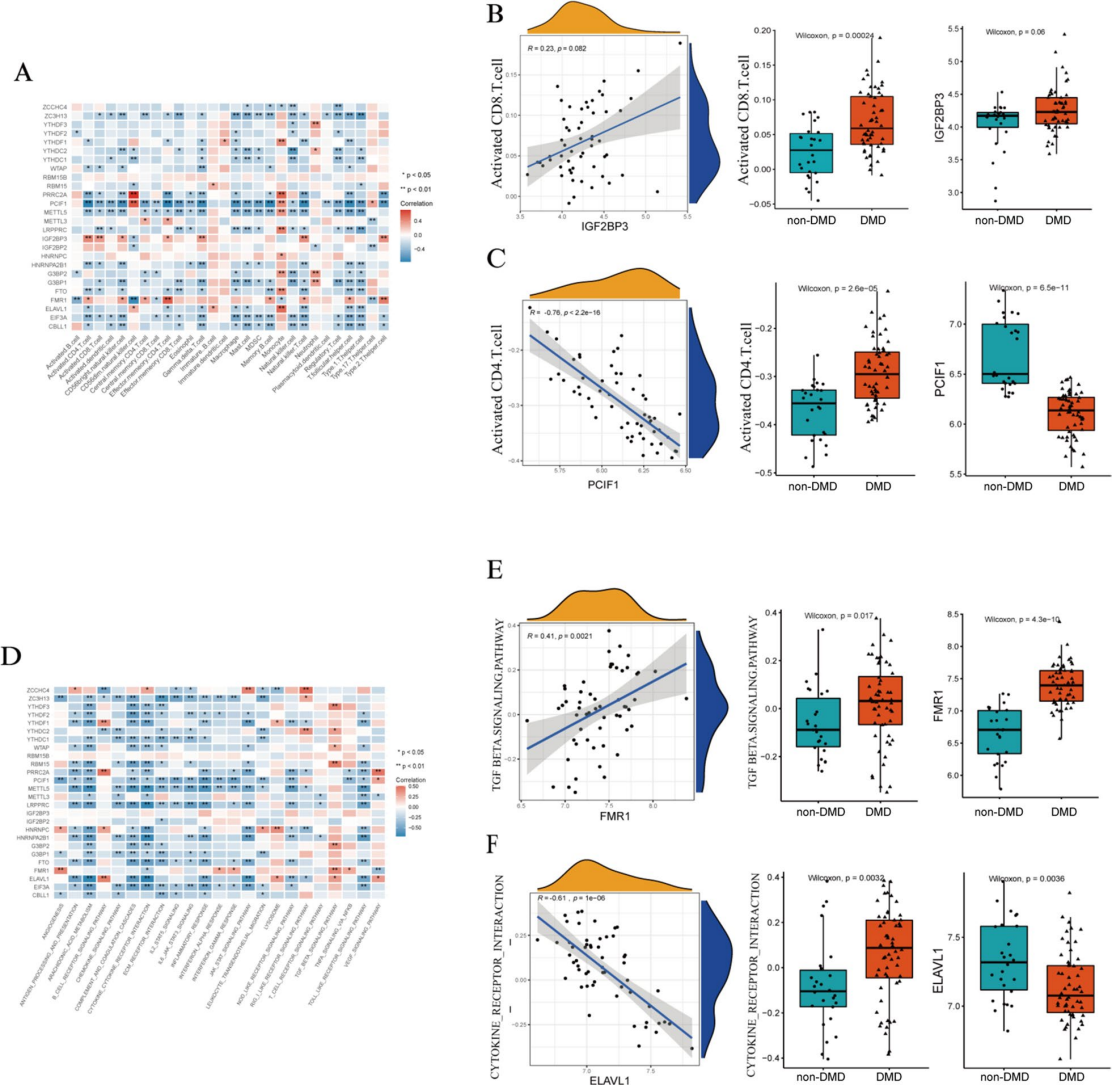
The relevance between m^6^A regulators and immune characteristics in DMD. **A** Heatmap showing the relationship between immune cells infiltration and m^6^A regulators. **B** The correlations between the expression values of IGF2BP3 and activated CD8^+^ T cells infiltration. **C** The correlations between the expression values of PCIF1 and activated CD4^+^ T cells infiltration. **D** Heatmap showing the relationship between the immune reaction gene-set and m^6^A regulators. **E** The correlations between the expression values of FMR1 and the activity of the TGF-β signaling pathway. **F** The correlations between the expression values of ELAVL1 and the activity of cytokine receptor interaction pathway. The expression levels, fraction status, or activity status are presented by a box-plot on the right panel of **B**, **C**,** E**, and **F**

The relevance between immune cell infiltration and the expression values of m^6^A regulators was estimated through correlation analysis in the muscle samples of DMD and a significant association was found (Fig. 4A, Additional file 7: Table S10). For instance, activated CD8^+^ T cell abundance was positively correlated with IGF2BP3 (Fig. 4B), while activated CD4^+^ T cell was negatively correlated with PCIF1 (Fig. 4C). Similarly, we found the main immune-related pathways have also been linked to the expression values of m^6^A regulators in DMD suggesting that these immune related pathways and m^6^A regulators interact with each other or have a regulatory relationship (Fig. 4D, Additional file 7: Table S11). For instance, the TGF-β signaling pathway was positively associated with several m^6^A regulators, while the Toll-like receptor signaling pathway was negatively correlated with multiple m^6^A regulators. Moreover, we found the m^6^A reader, FMR1 and ELAVL1, were highly associated with many immune response gene sets. As seen in Figs. 4E and F, FMR1 was positively related to the TGF-β signaling pathway; in turn ELAVL1 was negatively related to cytokine receptor interaction. Besides, the relevance between m^6^A regulators and HLA expression was analyzed (Additional files 6 and 7 Figure S6A, Table S12). The result indicated that ZCCHC4 and HLA-DOB were the most positively correlated pair (Additional file 6: Figure S6B), but the most negatively were HNRNPA2B1 and HLA-DOA (Additional file 6: Figure S6C).

### Consensus clustering of m^6^A regulators identified three types of patients with DMD

**Fig. 5:**
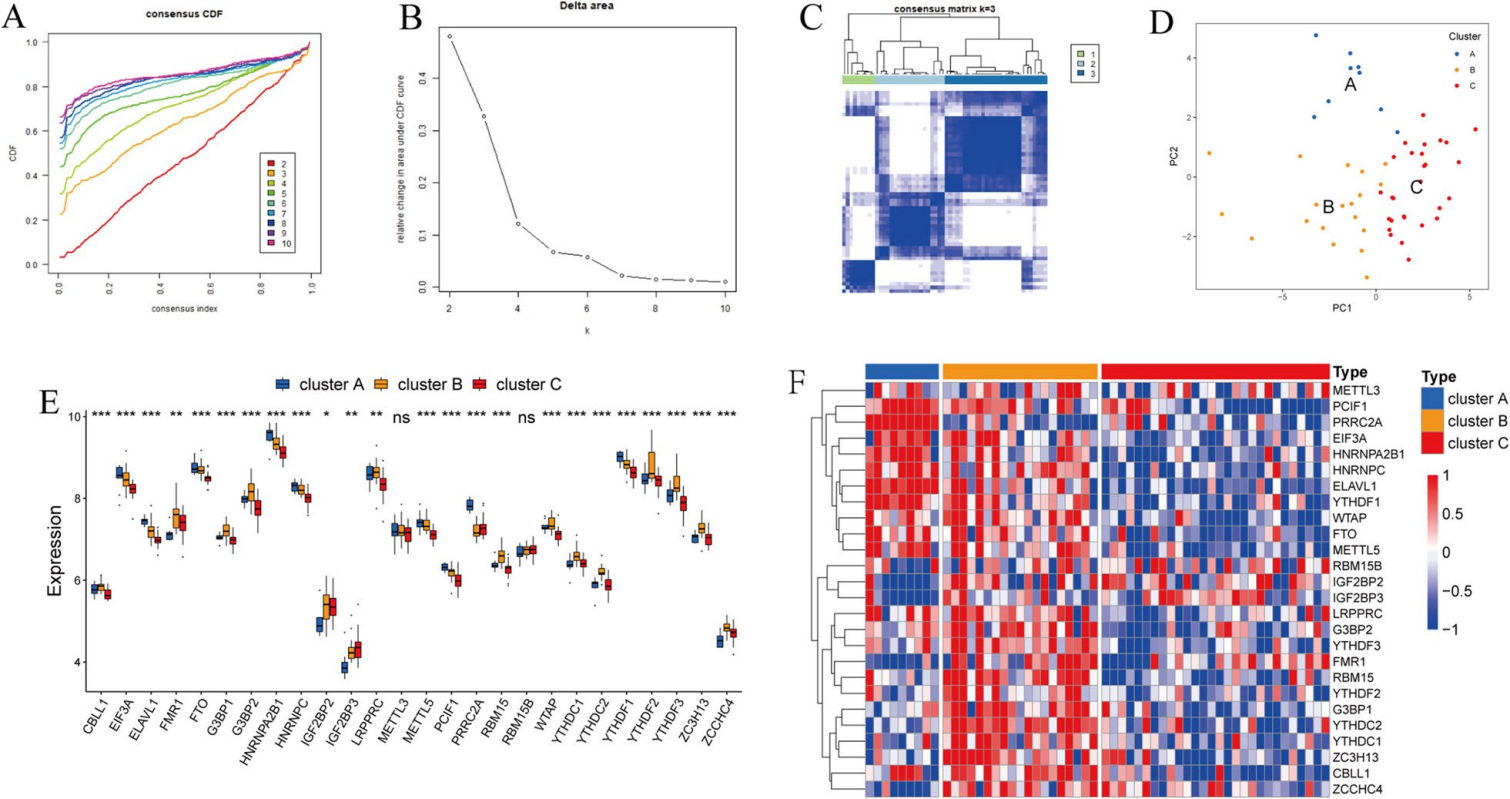
Consensus clustering of m^6^A regulators determined three DMD subtypes. **A** Consensus clustering cumulative distribution function (CDF) for k = 2 to 10. **B** The area under CDF for k = 2 to 10. **C** DMD patients were grouped into 3 clusters (k = 3). **D** PCA plot according to the transcriptome profiles of three m^6^A clusters. **E** and **F** The box plot and heatmap showing the expression values of m^6^A regulators in three clusters. *P < 0.05; **P < 0.01; ***P < 0.001

Consensus clustering was introduced to categorize patients with DMD into subgroups based on the expression levels of m^6^A regulators. With clustering stability increasing from k = 2 to k = 10, k = 3 was determined with appropriate clustering stability (Fig. 5A and B). Hence, DMD patients were clustered into three groups, including 9 samples in cluster A, 19 samples in cluster B and 28 samples in cluster C (Fig. 5C, Additional file 7: Table S13). PCA analysis further validated that the samples of DMD were separated into three non-overlapping clusters clearly (Fig. 5D). In addition, the expression differences of m^6^A regulators among the three cluster groups were evaluated and the distributions of m^6^A regulators' expression levels exhibit notable differences except for METTL3 and RBM15B (Fig. 5E, F).

### Characteristics analysis of immune microenvironment in distinct m^6^A clusters

**Fig. 6:**
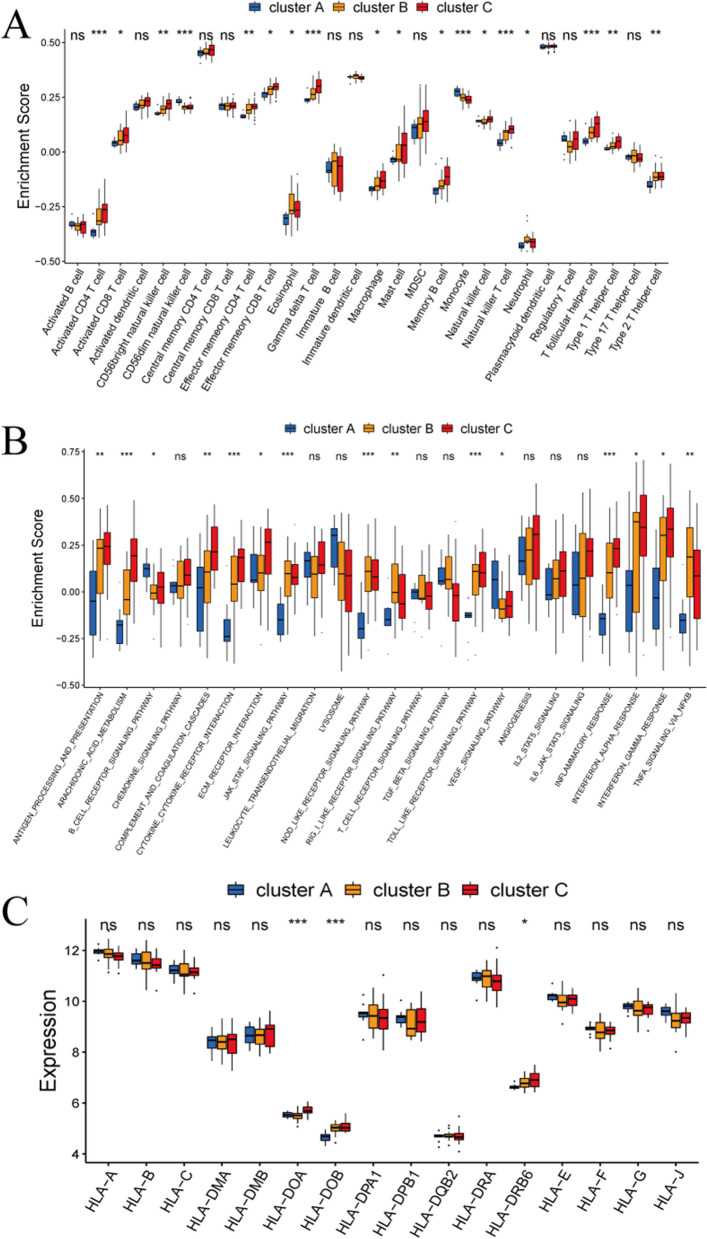
Characteristics analysis of immune microenvironment in different m^6^A clusters. **A** The abundance differences of immune cell infiltration in three m^6^A clusters. **B** The activity differences of immune reaction gene-sets in three m^6^A clusters. **C** The expression differences of HLA genes in three m^6^A clusters. *P < 0.05; **P < 0.01; ***P < 0.001

Infiltrated immunocytes were evaluated to characterize the immune infiltration among different m^6^A clusters. The proportion of 18/28 infiltrating immune cells was significantly heterogeneous in the different patterns (Fig. 6A). The abundance of most infiltrated immunocytes, including macrophages, activated CD4^+^ T cells, activated CD8^+^ T cells, and the natural killer cells were significantly higher in cluster C compared with cluster A or B. Immune response signaling pathways mediated by three clusters were also characterized (Fig. 6B). The result demonstrated that most of the immune pathways were activated in clusters B and C, while in a state of suppression in cluster A. In addition, the types of immune responses induced by cluster B and cluster C might be different. The immune reactions of ECM receptor interaction and cytokine receptor interaction were relatively more active in cluster C in contrast to TNF-α signaling via NF-κB which was stronger in cluster B. Moreover, the HLA gene expression showed a similar trend in these three patterns (Fig. 6C). Together, these data suggested the important role of m^6^A modification in shaping different immune microenvironments of DMD patients.

### Biological characteristics of m^6^A modification clusters

**Fig. 7:**
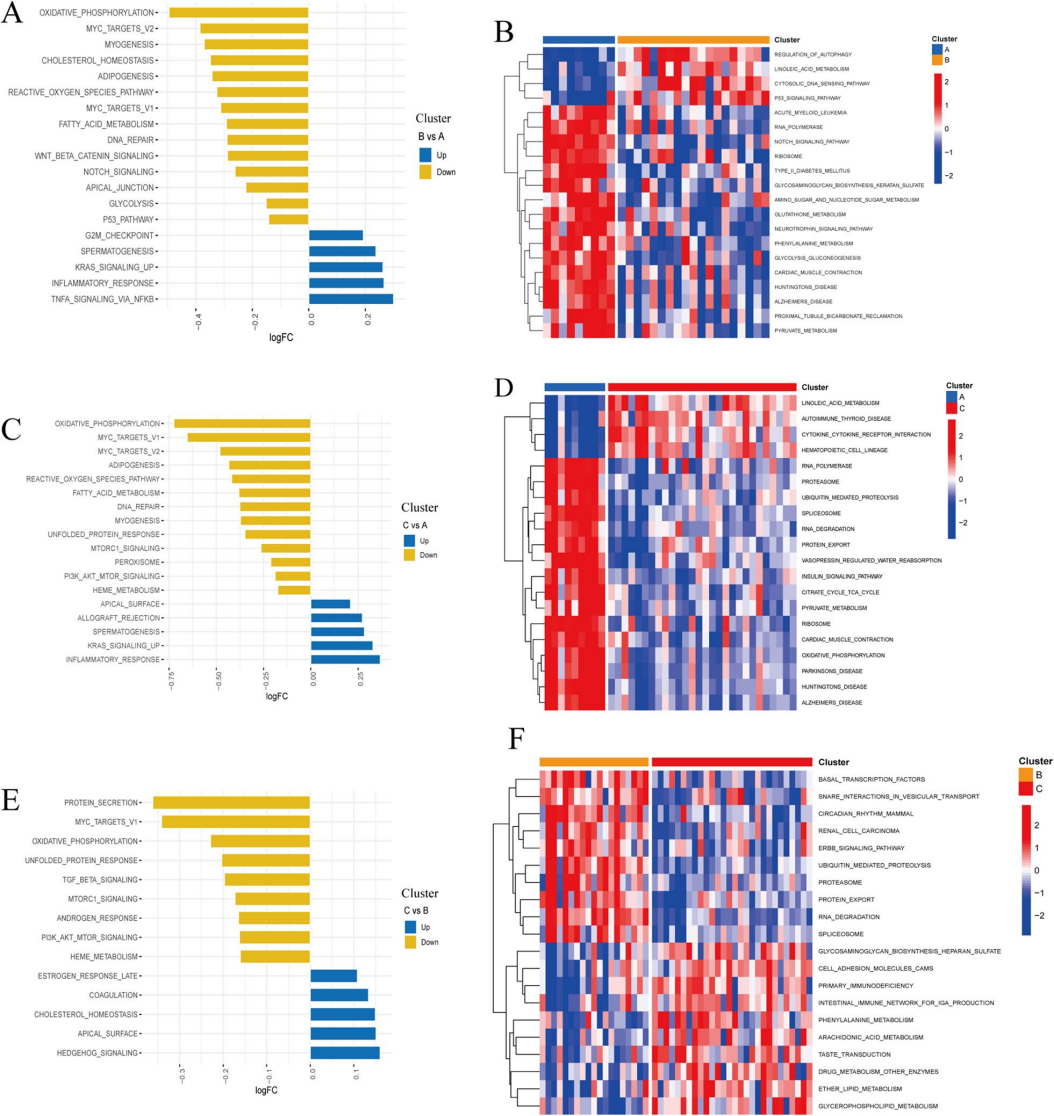
Biological characteristics of m^6^A modification clusters. The differences of HALLMARKS pathway (left) and KEGG pathway (right) enrichment score between each m^6^A modification cluster. **A** and **B**: cluster B vs. A, **C** and **D**: cluster C vs. A, **E** and **F**: cluster C vs. B

To evaluate the biological functions in distinct m^6^A modification patterns, we utilized GSEA to perform pairwise comparisons of the HALLMARKS and KEGG pathways among the three clusters. According to the FDR < 0.05, representative of hallmark gene sets were enriched, and principally are oxidative phosphorylation and myc target. Additionally, significant pathways on KEGG gene sets were explored, including linoleic acid metabolism, RNA degradation, and pyruvate metabolism. According to the enrichment features of the two gene sets, cluster A was negatively correlated with inflammatory response and allograft rejection compared with cluster B or C, suggesting the low immunity (Fig. 7A–D). Additionally, the enriched pathways numbers were almost identical in cluster B and cluster C (Fig. 7E, F).

### Identification of m^6^A regulators related genes in DMD

**Fig. 8:**
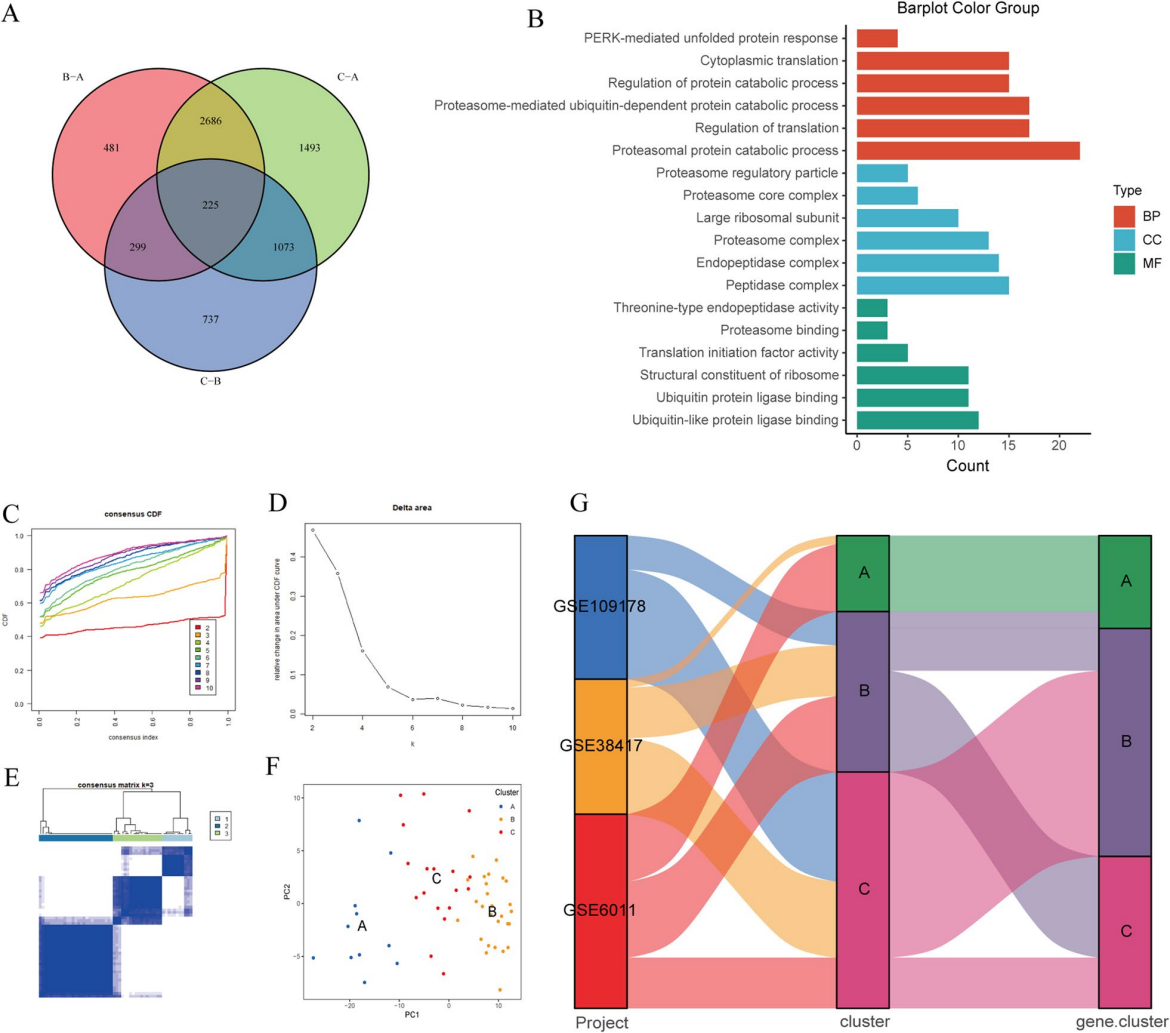
Identification and function analysis of m^6^A phenotype-related genes in DMD. **A** Venn diagram showing 225 m^6^A phenotype-related genes. **B** GO annotation of the biological features of m^6^A phenotype-associated genes. **C** The consistency clustering CDF curve for k = 2–10. **D** The area under the CDF for k = 2 to 10. **E** The m^6^A phenotype-related genes were divided into three distinct clusters (k = 3). **F** PCA plot based on the transcriptome profiles of 3 m^6^A phenotype-related genes clusters. **G** Alluvial diagram of three datasets of GEO in groups with m^6^A cluster and m^6^A geneCluster

To elucidate the mechanisms of genes participated in m^6^A regulator mediated regulation, we investigated differentially expressed genes (DEGs) associated with the m^6^A phenotype among three clusters. In total, 225 DEGs were determined as m^6^A phenotype-associated genes (Fig. 8A and Additional file 7: Table S14). Then, we performed GO enrichment analysis based on these DEGs and the results are illustrated in Fig. 8B. The biological process (BP) analysis showed the process of protein catabolic, regulation of translation, and cytoplasmic translation. Cellular component (CC) analysis principally included the peptidase complex, endopeptidase complex, and proteasome complex. Molecular function (MF) analysis revealed ubiquitin-like protein ligase binding, ubiquitin protein ligase binding, and structural constituent of ribosome. Furthermore, the consensus clustering was performed based on the expression of genes associated with the m^6^A phenotype. By choosing a k value of 3, three different clusters of DMD patients were determined, among which, A, B, and C contained 11, 18, and 27 samples, respectively (Fig. 8C–E). Furthermore, the result of PCA showed that DMD patients in the three clusters were identifiable (Fig. 8F). We also explored the distribution of samples in different datasets, m^6^A cluster and m^6^A related gene cluster (Fig. 8G). The result revealed that the patients in m^6^A cluster A belong to the m^6^A related gene cluster A group. For patients in m^6^A cluster B, the majority of patients belong to the m^6^A related gene cluster C group, and the remaining patients belong to the A or B group. Additionally, patients in m^6^A cluster C are categorized into two distinct m^6^A related gene subcategories B and C (Additional file 7: Table S15). This further suggested the three distinct m^6^A modification patterns existed in DMD samples.

## Discussion

It is widely known that deficiency of dystrophin in DMD results in a series of symptoms, including progressive inflammatory response and muscle damage. Inflammation is the major factor that contributes to skeletal muscle fibrosis and ultimately results in progressive muscle wasting, functional disability, and reduced lifespan [26]. Furthermore, the disordered immune microenvironment hampered the therapeutic response and clinical outcomes of many promising approaches aimed at restoring dystrophin (including stem cell transplantation, gene therapy, and exon skipping) [27, 28]. Thereby, it is biologically essential to have an in-depth understanding of the immunomodulatory mechanisms for developing new therapeutic strategies for DMD patients.

The present research first applied a comprehensive approach to characterizing the immune microenvironment in DMD skeletal muscle through bioinformatic analysis and experimental confirmation. By analyzing the expression data from DMD tissues and non-DMD muscle tissues by ssGSEA, we described the landscape of infiltrating immune cells in DMD skeleton muscle tissues and discovered the fraction of 26 immune cells was significantly altered. As the main infiltrating cells participated in dystrophin-deficient muscles [29], the increased infiltration of macrophages, CD4^+^ and CD8^+^ T cells was further validated by the FCM analysis in mdx and IHC in DMD patients. These results were in agreement with previous studies and suggested that both adaptive and innate immune cells may contribute to the pathogenesis of DMD [30, 31]. We also found the up-regulated immune-related pathways and altered HLA gene expression status in DMD skeleton muscle tissues, which further confirmed the complexity of immune microenvironmental changes in DMD.m^6^A RNA methylation is the most widespread pattern of post-transcriptional modification in eukaryotic. Previous studies have clearly shown that m^6^A can exert essential effects on regulating the immune system in a wide range of pathologies including tumorigenesis and viral infection [15, 17, 32]. However, to date, few studies have investigated the potential role of m^6^A modification in the immune microenvironment of DMD. We performed bioinformatics analyses to provide a picture of m^6^A modification in DMD immune microenvironment. The PPI network and expression correlation analyses revealed the close interactions among the m^6^A regulators which may help us to gain further insight into the regulatory mechanism of m^6^A modification. Of note, G3BP2 and YTHDF3 existed the most significant positive correlations. As one of the key components of stress granules (SGs), G3BP stress granule assembly factor 2 (G3BP2) is mainly mediated by the positive regulation of SG assembly and protein homooligomerization. Fu et al. confirmed the m^6^A-binding YTHDF proteins played an important role in SG formation. YTHDF1/3 depletion restricted SG formation and prevented the enrichment of mRNA signals in SGs [33]. Therefore, we speculate the interaction between G3BP2 and YTHDF3 may mediate the progression of DMD by involving in related oxidative stress response pathways. Besides, we investigated the expression levels of 26 m^6^A regulators and most of them were altered in the DMD group compared with the non-DMD group, suggesting that m^6^A regulators may be relevant to the pathology of DMD.

Furthermore, we established a m^6^A related diagnostic signature including 7 genes (FMR1, FTO, G3BP1, IGF2BP3, LRPPRC, YTHDC1, and ZCCHC4). The ROC curve analyses revealed that the m^6^A signature has a good performance to discriminate between non-DMD and DMD. For m^6^A writer, zinc finger CCHC domain-containing protein 4 (also known as ZCCHC4) is mainly involved in 28S rRNA methylation [34] and relevant to the fate of core cytokines in inflammatory bowel diseases [35]. For m^6^A reader, a recent study revealed that fragile X-linked mental retardation syndrome protein 1 (FMR1) knockout mice present with deficiencies in proinflammatory cytokine expression, specifically tumor necrosis factor-α expression and interleukin-6 in hippocampal [36]. Additionally, the mutation of FMR1 in the drosophila model led to a decrease in bacterial phagocytosis [37]. These shreds of evidence suggested that fragile X-linked mental retardation syndrome protein 1 (FMR1) could modulate the activity of immune system. It has been established that GTPase-activating protein SH3 domain-binding protein 1 (G3BP1, reader) can regulate the activation of the NF-κB pathway and type 1 interferon signaling, thereby affecting the immune response [38]. As NF-κB signaling is regarded as a crucial signaling pathway involved in the chronic inflammation status of dystrophic muscle, we speculate that G3BP1 may contribute to cellular damage and progression of DMD and represent a potential therapeutic target for DMD. m^6^A reader insulin-like growth factor 2 mRNA binding protein 3 (IGF2BP3) may affect prognosis in hepatocellular carcinoma by modulating the TGF-β signaling pathway [39]. LRPPRC, also named the leucine-rich PPR-motivated protein, is a member of the pentapeptide repeat (PPR) family. In antiviral immunity, deletion of LRPPRC expression results in increased activation of the IFN response [40]. YTHDF1 (reader) belongs to the YTH domain family. Silencing of YTHDC1 led to increased expression of M1 phenotypic markers, enhanced production of proinflammatory cytokines, and promoted migration of microglial [41]. For m^6^A eraser, fat mass and obesity associated protein (FTO) is an RNA demethylase and has been validated to participate in the regulation of muscle differentiation. New evidence is emerging that reduced FTO activity contributed to increased m^6^A methylation levels of IL-1β, IL-6 and TNF-α transcripts and aggravates inflammation in cardiomyocytes [42]. Therefore, these 7 m^6^A regulators may contribute to the progression of immune response and further research is warranted to investigate the roles of these signatures in DMD.

Next, we characterized the potential mechanisms underlying the regulation of m^6^A modification in the immune microenvironment of muscle tissues in DMD to search the possible immunotherapeutic target. As expected, we found that most m^6^A regulators are closely associated with the infiltration of immune cells in DMD. For example, the abundance of IGF2BP3 was positively relevant to infiltration of activated CD8^+^ T cells and PCIF1 expression had a negative relevance to activated CD4^+^ T cell infiltration which is consistent with what has been previously observed in thyroid carcinoma but contrary to the findings in kidney renal clear cell carcinoma [43]. Further research is warranted to delineate the involved biological processes. Similarly, there was a strong association between the m^6^A regulators and the activity of the immune pathways, suggesting a crucial role of m^6^A modification in the regulation of immune responses. Notably, TGF-β signaling pathway which is involved in chronic inflammatory response and fibrosis in DMD [44, 45], is associated with a variety of m^6^A regulators. In fact, it has been established that METTL3-METTL14-WTAP complex interacts with TGF-β pathway through the SMAD2/3 interactome [46]. In addition, a recent study revealed that the m^6^A reader YTHDF3 can influence TGF-β signaling pathway by mediating peroxiredoxin 3 translation in liver fibrosis [47]. Our data discovered the close relationship between the TGF-β signaling pathway and FMR1 in DMD which has not been reported previously and may provide a new insight into the regulation of this signaling pathway.

The role of epigenetic modifications has been increasingly appreciated based on its potentially relevant implications in identifying homogeneous groups of patients with different characters, which can advance our understanding of the pathophysiology and formulate individualized therapeutic strategies [48]. More recently, molecular techniques such as genotyping chips and next generation sequencing (NGS) have enabled the rapid and cost-efficient studying of epitype [49]. By analyzing the expression data from 56 DMD samples, we conducted consensus clustering and determined three DMD subtypes (clusters A/B/C) with diverse immune characteristics based on the expression of m^6^A regulators. We found that m^6^A cluster C presented the highest infiltration of immune cells and strongest immune responses than cluster A and cluster B. In addition, the immune related pathways affected by different m^6^A modification clusters varied greatly. The substantial differences exist in the immune microenvironment among the three clusters may lead to m^6^A different responses to therapy and have different outcomes. By identifying different m^6^A regulator-based expression patterns, it will be possible to develop more effective and targeted interventions to improve the prognosis of patients with DMD.

Although our work included a relatively large sample size by integrating GEO datasets to discover the role of m^6^A in the immune microenvironment of DMD, some limitations need to be considered. First, we investigated the immune cell infiltration through ssGSEA and chose the major infiltrating cell populations (macrophages, CD4^+^ and CD8^+^ T cells) for FCM and IHC validation. Further studies are still needed to characterize the infiltrating immunocytes and their exact mechanisms more thoroughly in DMD patients. Second, as our results are mainly based on the bioinformatic analysis of datasets, additional validation will likely need to be derived from experimental studies. In addition, the level of gene expression slightly differed between the training and validation dataset. This may be due to the inter-individual differences and smaller sample size in the GEO dataset, which inevitably affects the accuracy of results.

## Conclusion

In this work, we characterized the overall landscape of the immune microenvironment in the skeletal muscle tissues of DMD and preliminary investigated the relevance between m^6^A regulators and DMD immune microenvironment. A diagnostic model involving seven m^6^A regulators was established with a well-performed risk score. Furthermore, three DMD subtypes (cluster A/B/C) were obtained with different immune microenvironmental characteristics through consensus clustering. The comprehensive analyses of the DMD m^6^A modification pattern may enhance our understanding of the immunomodulatory mechanisms in DMD and provide novel potential strategies for DMD therapy.

## Supplementary Information

**Additional file 1: Figure S1.** PCA plots for three datasets. **A** PCA plots for three datasets without processing. **B** PCA plots for three datasets with removing batch effects.

**Additional file 2: Figure S2.** Gene set enrichment analysis pathways involved in DMD. **A** antigen processing and presentation, **B** intestinal immune network for IGA production, **C** allograft rejection, **D** graft versus host disease, **E** complement and coagulation cascade, **F** leukocyte transendothelial migration.

**Additional file 3: Figure S3.** The activity differences of immune reaction gene-set between DMD patients and non-DMD controls. The overall landscape of immune reaction levels between DMD patients and non-DMD controls. **B** Violin diagrams showing the scores for the immune reaction levels of DMD patients and non-DMD controls. * P < 0.05; ** P < 0.01; *** P < 0.001; ns, no significance

**Additional file 4: Figure S4.** The box-plot shows the expression difference of HLA genes in DMD patients and non-DMD control. * P < 0.05; ** P < 0.01; *** P < 0.001; ns, no significance.

**Additional file 5: Figure S5.** The gene expressions of key m^6^A regulators were validated using an independent dataset. * *P* < 0.05; ** *P* < 0.01; *** P < 0.001; ns, no significance.

**Additional file 6: Figure S6.** The correlation between m^6^A regulators and HLA genes in DMD. **A** Heatmap showing the correlations between HLA genes and m^6^A regulators. **B** The relationship between HLA-DOB and ZCCHC4. **C** The relationship between HLA-DOA and HNRNPA2B1. The expression levels of genes are presented by a box plot on the right panel of B and C. * P < 0.05; ** P < 0.01; *** P < 0.001; ns, no significance.

**Additional file 7: Table S1.** Clinical background information of the human participants. **Table S2.** Difference on immune related gene sets between healthy and DMD samples. **Table S3.** Difference on immune cell infiltration fraction between healthy and DMD samples. **Table S4.** Difference on HLA gene expression between healthy and DMD samples. **Table S5.** m^6^A RNA methylation regulators. **Table S6.** Expression diversity of m^6^A regulators. **Table S7.** Correlations between m^6^A regulators and transcription factors. **Table S8.** Univariate logistic regression of m^6^A regulators. **Table S9.** LASSO regression coefficient. **Table S10.** Correlation between m^6^A regulators and immune related gene sets. **Table S11.** Correlations between m^6^A regulators and immune cell infiltration fraction. **Table S12.** Correlation between m^6^A regulators and HLA gene. **Table S13.** m^6^A modification patterns. **Table S14.** m^6^A related genes. **Table S15.** Change of sample attributes.

## Data Availability

Raw data in the study can be available from the corresponding author on reasonable request.
