# Supplementary figures and images for: Comprehensive analysis of m6A regulators characterized by the immune microenvironment in Duchenne muscular dystrophy
Source: J Transl Med. 2023 Jul 11;21:459. doi: 10.1186/s12967-023-04301-5 (PMC10334523; doi:10.1186/s12967-023-04301-5)

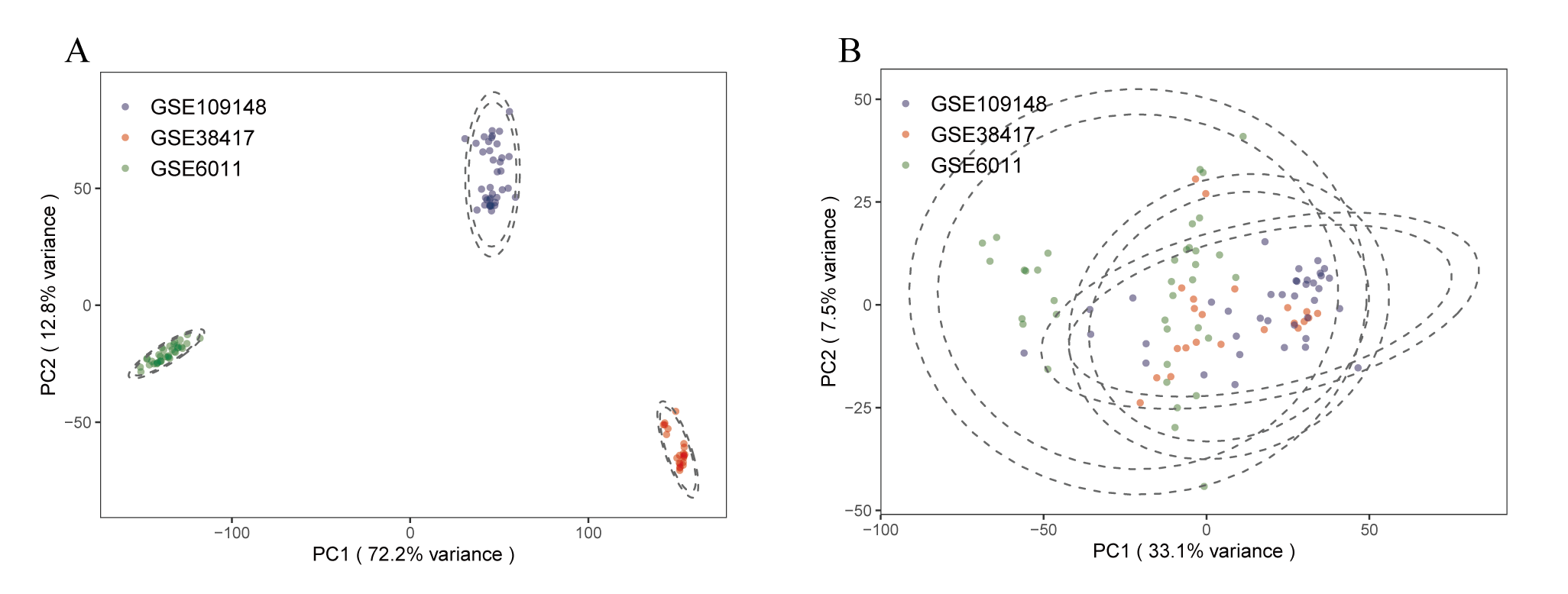

Supplement: Supplementary file 1 — Additional file 1: Figure S1. PCA plots for three datasets. A PCA plots for three datasets without processing. B PCA plots for three datasets with removing batch effects. [file 12967_2023_4301_MOESM1_ESM.tif]

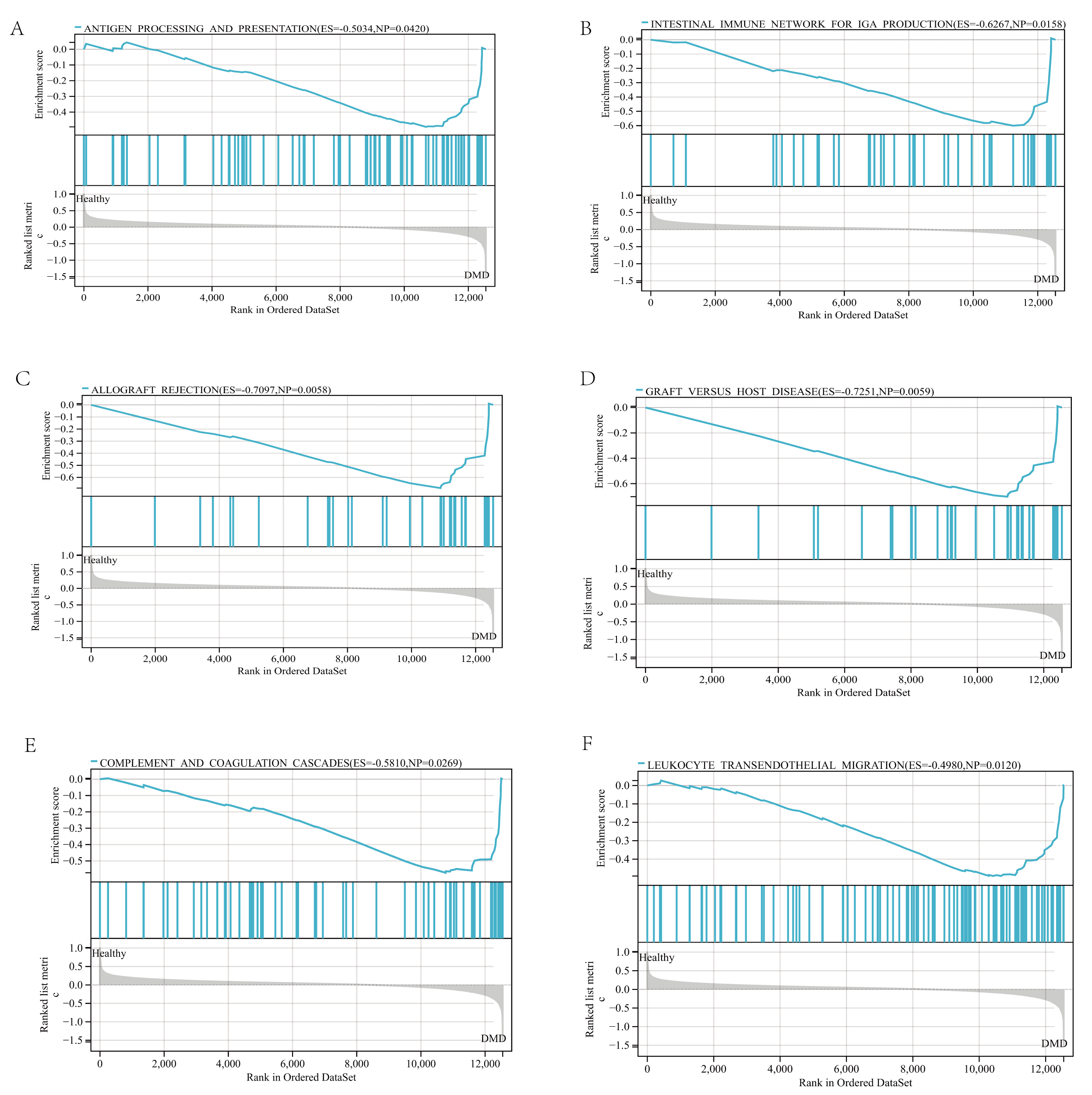

Supplement: Supplementary file 2 — Additional file 2: Figure S2. Gene set enrichment analysis pathways involved in DMD. A antigen processing and presentation, B intestinal immune network for IGA production, C allograft rejection, D graft versus host disease, E complement and coagulation cascade, F leukocyte transendothelial migration. [file 12967_2023_4301_MOESM2_ESM.tif]

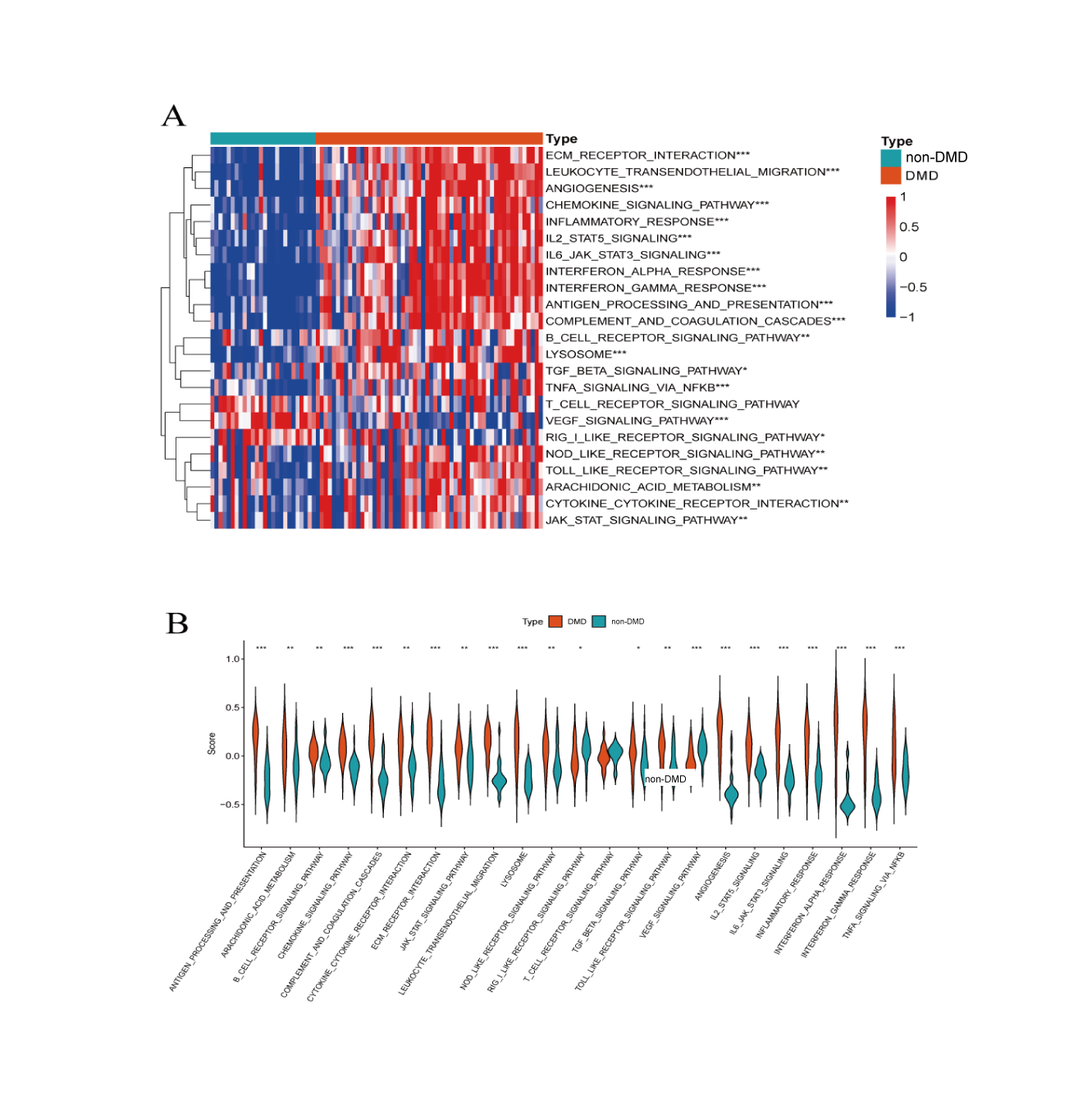

Supplement: Supplementary file 3 — Additional file 3: Figure S3. The activity differences of immune reaction gene-set between DMD patients and non-DMD controls. The overall landscape of immune reaction levels between DMD patients and non-DMD controls. B Violin diagrams showing the scores for the immune reaction levels of DMD patients and non-DMD controls. * P < 0.05; ** P < 0.01; *** P < 0.001; ns, no significance [file 12967_2023_4301_MOESM3_ESM.tif]

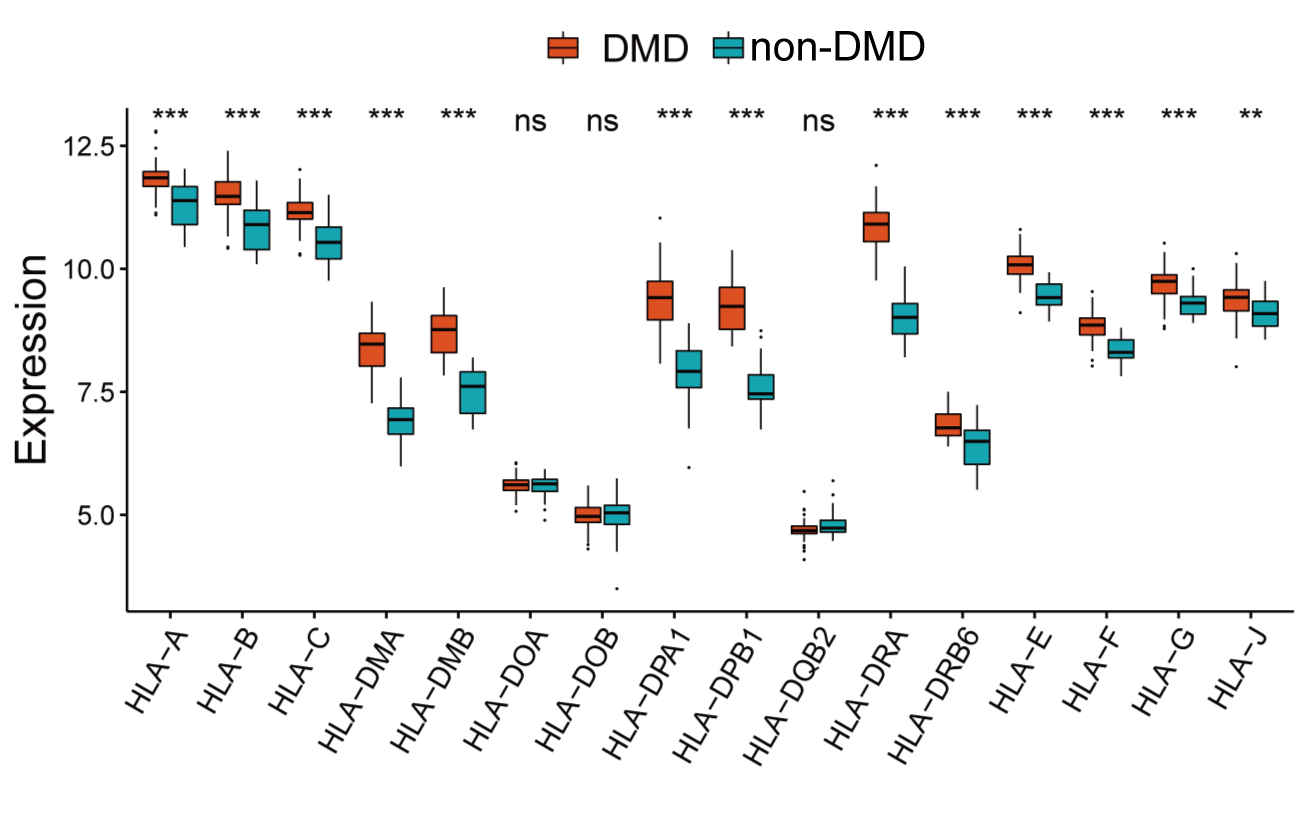

Supplement: Supplementary file 4 — Additional file 4: Figure S4. The box-plot shows the expression difference of HLA genes in DMD patients and non-DMD control. * P < 0.05; ** P < 0.01; *** P < 0.001; ns, no significance. [file 12967_2023_4301_MOESM4_ESM.tif]

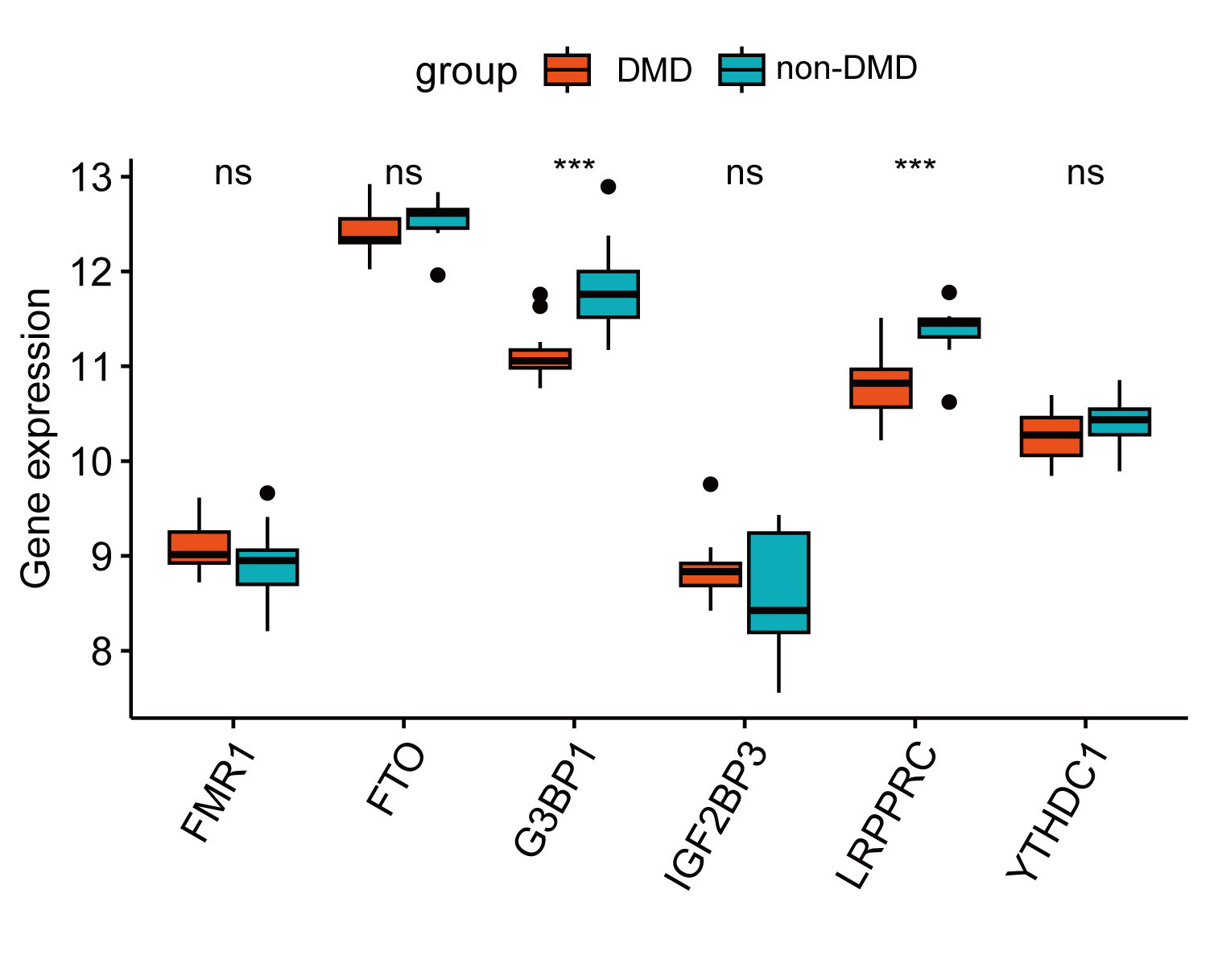

Supplement: Supplementary file 5 — Additional file 5: Figure S5. The gene expressions of key m6A regulators were validated using an independent dataset. * P < 0.05; ** P < 0.01; *** P < 0.001; ns, no significance. [file 12967_2023_4301_MOESM5_ESM.tif]

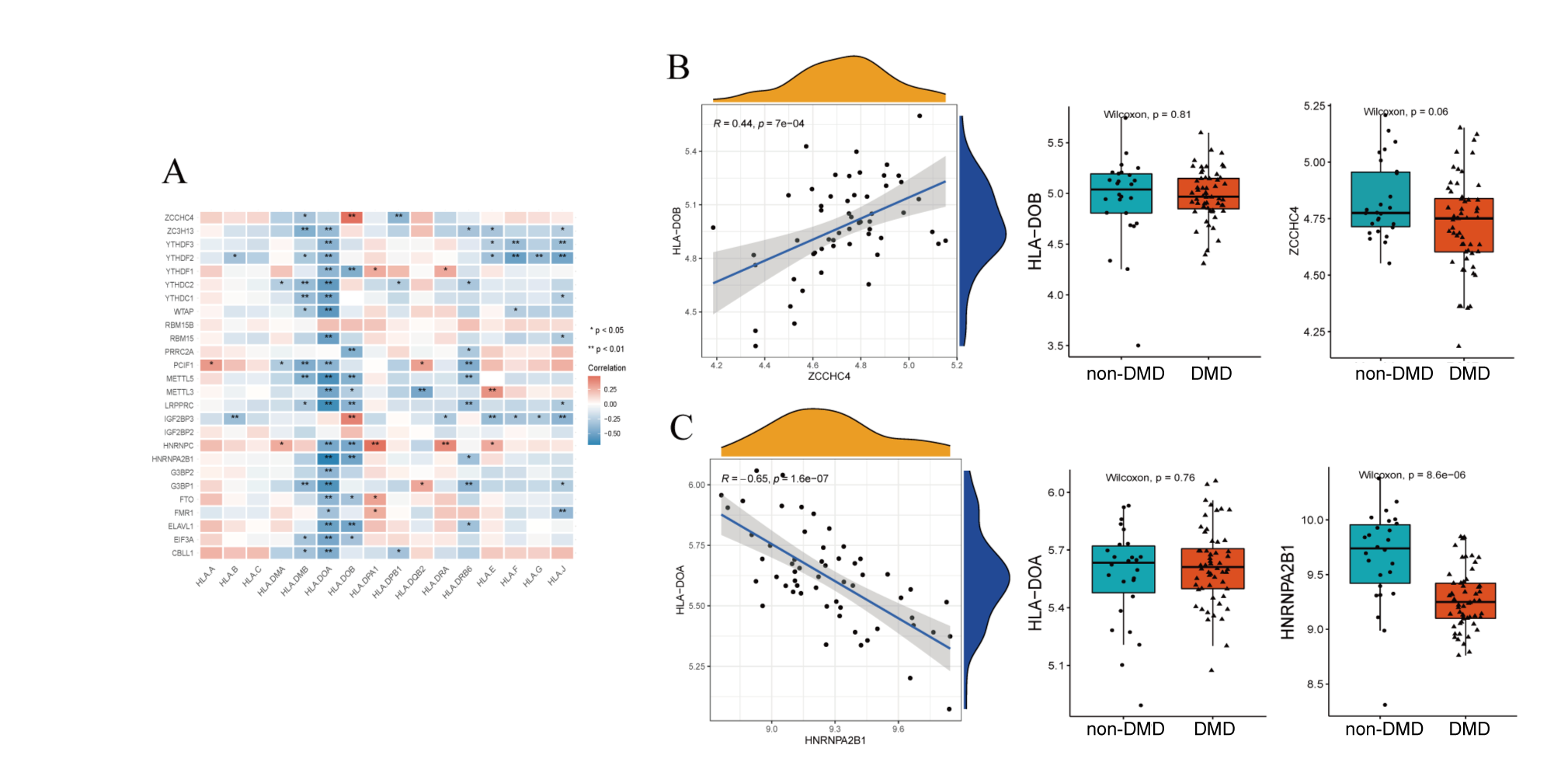

Supplement: Supplementary file 6 — Additional file 6: Figure S6. The correlation between m6A regulators and HLA genes in DMD. A Heatmap showing the correlations between HLA genes and m6A regulators. B The relationship between HLA-DOB and ZCCHC4. C The relationship between HLA-DOA and HNRNPA2B1. The expression levels of genes are presented by a box plot on the right panel of B and C. * P < 0.05; ** P < 0.01; *** P < 0.001; ns, no significance. [file 12967_2023_4301_MOESM6_ESM.tif]
